# Circulating miRNAs act as potential biomarkers for asthma

**DOI:** 10.3389/fimmu.2023.1296177

**Published:** 2023-12-19

**Authors:** Guang Hu, Yujie Du, Manying Xie, Rongchang Chen, Fei Shi

**Affiliations:** ^1^ Department of Infectious Disease, Shenzhen People’s Hospital (The Second Clinical Medical College, Jinan University; The First Affiliated Hospital, Southern University of Science and Technology), Shenzhen, Guangdong, China; ^2^ Intervention Department, Shenzhen People’s Hospital (The Second Clinical Medical College, Jinan University; The First Affiliated Hospital, Southern University of Science and Technology), Shenzhen, Guangdong, China; ^3^ Key Laboratory of Shenzhen Respiratory Diseases, Institute of Shenzhen Respiratory Diseases, Shenzhen People’s Hospital (The Second Clinical Medical College, Jinan University; The First Affiliated Hospital, Southern University of Science and Technology), Shenzhen, Guangdong, China

**Keywords:** biomarker, circulating miRNA, asthma, light, diagnosis

## Abstract

**Background:**

Identification of new clinical markers contributes to a better understanding of the pathogenesis of asthma. Considering the crucial role of LIGHT in asthma, it may become a potential target for asthma. The aim of current study was to determine if circulating microRNAs (miRNAs) targeting LIGHT may be used as diagnostic biomarkers to distinguish asthma.

**Methods:**

Blood serum from a cohort of 60 subjects, including 20 cases with mild asthma, 20 cases with moderate-to-severe asthma, and 20 healthy controls were included. Serum was analyzed for circulating miRNAs profiles through miRNAs microarray. Real Time PCR was conducted to verify the results of miRNA microarray. Correlations between circulating miRNAs targeting LIGHT and clinical characteristics were investigated.

**Results:**

A total of 365 miRNAs were differentially expressed in asthma patients. Among them, miR-107 and miR-140-5p were found to target LIGHT, and varied in asthmatics. Additionally, miR-107 and miR-140-5p expressions were positively correlated with the absolute value of peripheral eosinophils. Finally, miR-140-5p and miR-107 were demonstrated to have good diagnostic efficacy for asthma (AUC= 0.8667 and 0.9400) with good sensitivity (0.8000 and 0.8667,respectively) and specificity (0.8667 and 0.867). Thus, circulating miRNAs expressed differentially between healthy control and asthma patients.

**Conclusion:**

Plasma miR-140-5p and miR-107 can be used as diagnostic biomarkers to distinguish patients with asthma from healthy control, and may take part in asthma pathogenesis by negatively regulating LIGHT. Further research was needed to evaluate their roles as potential biomarkers in the diagnosis of asthma.

## Introduction

Bronchial asthma is characterized by chronic inflammation in airway, accompanied by airway hyperresponsiveness and obstruction with an increase over several decades. Existing treatments or drugs often cannot prevent the occurrence of asthma and are not completely effective (1). It has become a vital direction of asthma research to find effective intervention targets.

LIGHT (lymphotoxin-related inducible ligand that competes for glycoprotein D binding to herpes virus entry mediator on T cells, also known as TNFSF14) has been identified as a pathogenic threat in various inflammatory diseases such as asthma (2). It promotes a type 2 inflammatory response by binding to the herpes virus entry mediator (HVEM) on CD4^+^ T cells, and facilitates airway remodeling via inducing extracellular matrix protein deposition and proliferation. According to our recent study (3), the expressions of LIGHT and its legend HVEM in the airway were increased in mice model of asthma, while inhaled glucocorticoid improved airway inflammation by partly suppressing LIGHT in the airway. Therefore, LIGHT might be a potential target for airway inflammation and remodeling of asthma.

MicroRNAs are RNAs of about 18-24 nucleotides in length, which play a crucial part in regulating gene expression at the post-transcriptional level (4). It has been recognized that miRNAs can be used as biomarkers for a variety of diseases due to their stability in serum, so we conduct the study to identify and screen the profile of circulating miRNAs targeting LIGHT in asthma.

## Materials and methods

### Study subjects

A total of 20 cases with mild asthma (symptoms: cough, soft wheeze, minor difficulty breathing, no difficulty speaking in sentences), 20 cases with moderate-to-severe asthma (symptoms: persistent cough, loud wheeze, obvious difficulty breathing, very distressed, speak short sentences or only a few words in one breath, pale and sweaty), and 20 age- and sex-matched healthy subjects were included in this case-control study. All asthma subjects were admitted to Shenzhen People’s Hospital from to May 2017 to April 2018.

40 cases met the guidelines and were included while 20 cases were excluded. Our study was composed of two parts: (1) Part 1 (**Supplementary Table 1**): Mild asthma group (n=5), moderate-to-severe asthma group (n=5), and healthy control group (n=5) for microRNAs microarray and primary Real-Time PCR; (2) Part 2 (**Supplementary Table 2**): Mild asthma group (n=15), moderate-to-severe asthma group (n=15), and healthy control group (n=15) for the validation of Real-Time PCR. The diagnostic criteria for asthma were based on the GINA guidelines, and the degree of asthma severity was determined by GINA guidelines at the time of recruitment. The exclusion criteria on for asthma patients included chronic obstructive pulmonary disease, interstitial lung disease, tuberculosis, tumor, and other systemic diseases affecting the respiratory system. Spirometry (SensorMedicsV6200), Fractional exhaled nitric oxide (FeNO, NIOXMINO, Sweden), differential cell counts, and serum total IgE were measured in individuals with asthma. (**Supplementary Table 3**) All participants were non-smokers or former smokers with smoking cessation of at least 10 years and a maximum of 10 pack years.

### Total RNA extraction

Total RNA was extracted from 250 μL of blood samples by using TRIzol LS Reagent (Invitrogen), Chloroform (Shanghai chemical reagent limited company), Isopropanol (Shanghai chemical reagent limited company), and absolute alcohol (Shanghai chemical reagent limited company). The RNA quality was detected by Nanodrop 1000 spectrophotometer (Agilent Technology, USA).

1) 750 μL TRIzol LS Reagent and 20 μL glacial acetic acid were added in 250 μL plasma samples in 1.5 mL markered tubes.
2) Mix them vigorously by vortex for 15 sec. Allow the mixture to stand for 2-3 min at room temperature.
3) Centrifuge at 10,000g in a centrifuge for 10 min at 4°C.
4) Transfer the upper clear phase to a fresh tube.
5) Add 0.5 mL of isopropanol and 5 μL glycogen in the clear phase. Mix vigorously by vortexing. Allow the mixture to stand for 10 min at room temperature.
6) Collect the precipitated RNA by centrifugation at 10,000g in a centrifuge for 10 min at 4°C.
7) Wash the isopropanol pellet by vortexing with 75% ethanol and centrifuge at 10,000g in a centrifuge for 10 min at 4°C.
8) Dry it and then resuspend the pellet with DEPC water.

### MiRNAs microarray analysis

The extracted total RNA was quantified, labeled, and hybridized using the Agilent miRNA Complete Labeling and Hyb Kit in conjunction with the Agilent Human miRNA microarray, performed by Cnkingbio Biotechnology Corporation (Beijing, China) (5).The gene expression profile data was extracted using Agilent Feature Extraction Software (11.0.1.1) and analyzed using Agilent GeneSpring GX 12.1 (Agilent Technologies). Transcripts that exhibited P ≤ 0.05 or fold changes greater than or equal to two were regarded as differential expressions. MiRNAs targeting LIGHT gene were predicted using TargetScan software by comparing differentially expressed miRNAs from healthy control, mild asthma subjects, and moderate-to-severe asthma subjects.

### Bioinformatics analysis of differentially expressed miRNAs

Genes targeted by miRNAs were predicted using the miRecords database. Consequently, GO enrichment and KEGG pathway enrichment analysis were performed basing on the differentially targeted genes by DAVID (2021).

### Real-time PCR to validate miRNAs expression

Real-Time PCR was performed to estimate the expression profile of miRNAs. The extracted RNA samples were transcribed by using the GoScript™ Reverse Transcriptase kit. FastStart Universal SYBR Green Master (Roche) was utilized to perform Real-Time PCR with cDNA as the template in a 10μL reaction system according to the manufacturer’s protocol via qPCR instruments (Bio-Rad’s CFX96 real-time system). The entailed program was preheating at 95 °C for 10 min, denaturing at 95 °C for 15 s for 39 cycles, annealing and extending at 60°C for 1 min. The results of Real-Time PCR of miR-140-5p and miR-107 correlated with or without clinical indexes were performed with pairwise comparison by Kruskal-Wallis test and a linear correlation regression.

### Statistical analysis

Prism 9 software (GraphPad Software) were utilized to analyze and present the results. The comparative cycle threshold method (2−ΔΔCt) was utilized to estimate the expression profile of miRNAs, in which U6 was an internal control. One way ANOVA or t Kruskal-Wallis test was utilized to calculate the difference in miRNAs expression profile. Receiver Operating Characteristic Curve (ROC curve) was employed to assess the diagnostic efficacy of the diagnostic model. *P*<0.05 represents a statistically significant difference.

## Results

### Clustering analysis of miRNA from recruited subjects through microRNAs microarray

**Figure 1:**
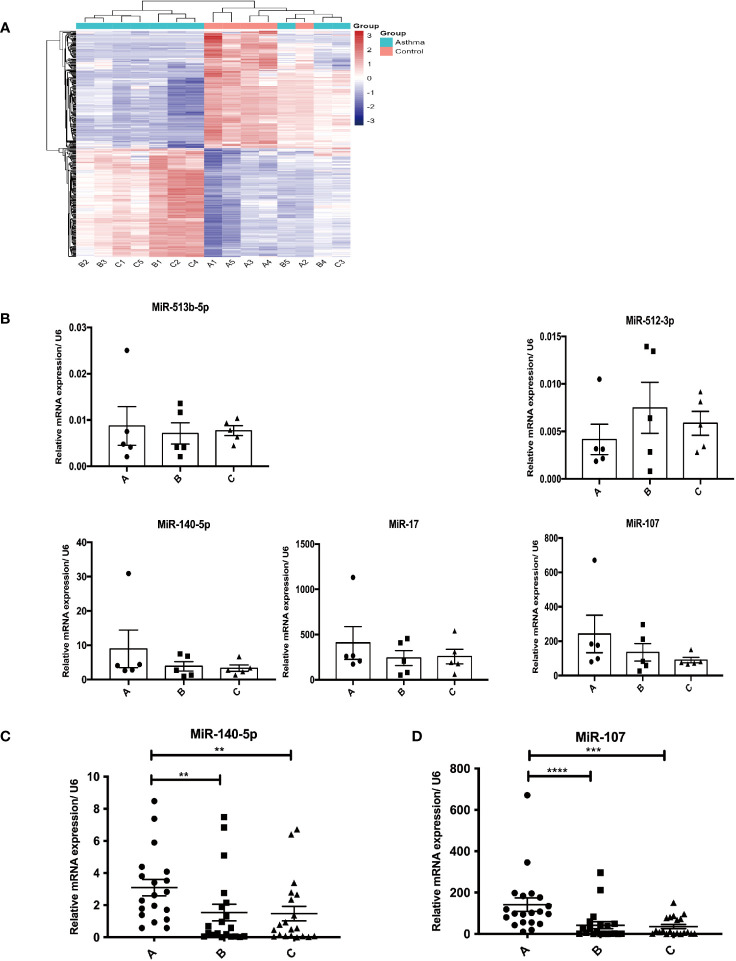
Circulating miRNAs screening and validating. **(A)** Heatmap analysis of miRNA profiles from three groups through miRNA microarray (A1-A5, B1-B5, and C1-C5 representing 5 samples from healthy control group, mild asthmatic patient group and moderate to severe asthmatic subject group respectively). **(B)** Primary validation of screened miRNA targeting LIGHT **(C)**. **(D)** Secondary identification of selected miRNA (**C** for miR-140-5p; **D** for miR-107). A–C in **Figure 1** representing healthy control group, mild asthmatic patient group and moderate to severe asthmatic subject group respectively. **p<0.01; ***p<0.001; ****p<0.0001.

The same type of samples can often appear in the same cluster through clustering, while the clustering of the sample in the same cluster generally shares similar biological characteristics. The results of the clustering analysis of miRNAs were shown with a heatmap of the microarray expression profile (GSE22284) (**Figure 1A**), where each column represented a miRNA and each row represented one sample. The results of clustering analysis of miRNA suggested that there were differences in miRNA expression profile among the healthy control group, mild asthma group, and moderate-to-severe asthma group. Red meant up-expressed miRNAs while green meant down-expressed miRNAs.

### Identifying and screening differentially expressed miRNAs targeting LIGHT

It was found that the miRNAs expressions of two asthma groups showed significant differences (The threshold for determination was a 2-fold or greater difference in miRNAs expression levels between groups) when compared with the healthy control group. According to the results of miRNAs microarray, there was a total of 176 miRNAs (eg. miR-6785-5pa, miR-4428, miR-6893-5p, miR-512-3p, miR-513b-5p, miR-5691) up-regulated, and 189 miR NAs (eg. miR-26b-5p, miR-107, miR-140-5p, miR-17-5p) down-regulated. The screened results of miRNAs were listed in **Supplementary Tables 4** and **5**.

Moreover, six members of the cluster of differentially expressed miRNAs, including miR-512-3p, miR-513b-5p, miR-5691, miR-107, miR-140-5p, and miR-17-5p, were found to target LIGHT by utilizing TargetScan software (**Supplementary Table 6**).

### Validating differentially expressed miRNAs targeting LIGHT

The primary Real-Time PCR demonstrated that expression of miR-513b-5p tended to increase while expressions of miR-140-5p, miR-17 and miR-107 tended to decrease in asthma groups in comparison with healthy controls (**Figure 1B**). The change trend of these four miRNAs were same as the results of miRNAs microarray. However, the expression of miR-512-3p was testified to be elevated in asthma patients by Real-Time PCR, which was contrary to the results of miRNAs microarray (**Supplementary Table 7**).

In addition, we increased the sample size to revalidate the results by secondary Real-Time PCR. The expressions of miR-140-5p and miR-107 was significantly decreased in asthma subjects when compared with healthy controls (P=0.0054, <0.0001, respectively), which was consistent with the results of primary Real-Time PCR (**Figures 1C, D**).

### Bioinformatics analysis of miR-140-5p and miR-107

The target genes of miR-140-5p and miR-107 were predicted by utilizing the miRecorcds database. Our findings indicated that there were 32,419 and 32,969 genes regulated by miR-140-5p and miR-107 respectively. To reduce the false positive rate, we selected genes predicted with four commonly used databases by researchers as target genes, which had 346 and 687 genes targeted by miR140-5p and miR-107, respectively.

GO functional enrichment analysis of screened genes targeted by miR140-5p and miR-107 were performed by DAVID and Prism software.

**Figure 2:**
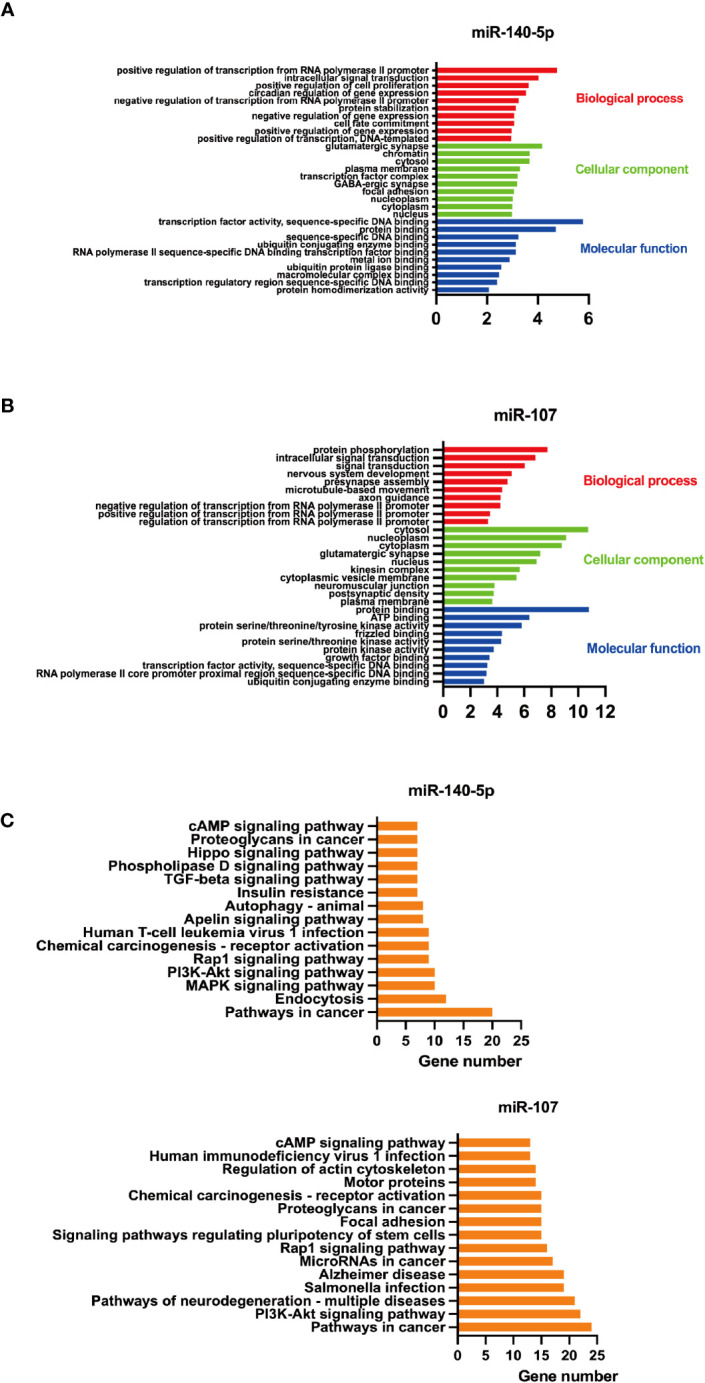
GO functional enrichment and KEGG pathway enrichment analysis of screened miRNAs. A+B GO functional enrichment analysis of screened miRNAs, **(A)** for miR-140-5p and **(B)** for miR-107.C+D. KEGG pathway enrichment analysis of these two miRNAs, **(C)** for miR-140-5p and **(D)** for miR-107.

The top 10 enriched GO terms were shown in **Figures 2A, B**. The screened genes targeted by miR-140-5p were mainly involved in biological processes, including “positive regulation of transcription from RNA polymerase II promoter”, “positive regulation of cell proliferation”, “negative regulation of gene expression”. “glutamatergic synpase”, “chromatin” ranked high in category of cellular component while “transcription factor activity, sequence-specific DNA binding” and “protein binding” were the primary enriched molecular function category (**Figure 2A**). In addition, the targeted genes by miR-107 play roles in biological processes, such as “protein phosphorylation”, “Intracellular signal transduction”, and “microtube-based movement”. In the category of cellular component, “cytosol”, “nucleoplasm”, and “cytoplasm” ranked the highest. while “protein binding” and “ATP binding” were the primary enriched molecular function (**Figure 2B**).

Thereafter, we utilized DAVID to predict KEGG pathways targeted by miR-140-5p and miR-107. It was found that miR-140-5p may play a role in MAPK, Rap1, cAMP, PI3K-Akt, and Apelin signaling pathways, which are related to a variety of functions including insulin resistance and macrophage function. Meanwhile, miR-107 may take a part in the signaling pathway of PI3K-Akt, Rap1, cAMP, and focal adhesion. These target genes were related to function regulation of stem cell, and associated with cancer (**Figures 2C, D**).

### MiR-140-5p and miR-107 expression level of three groups of subjects and clinical indexes

**Figure 3:**
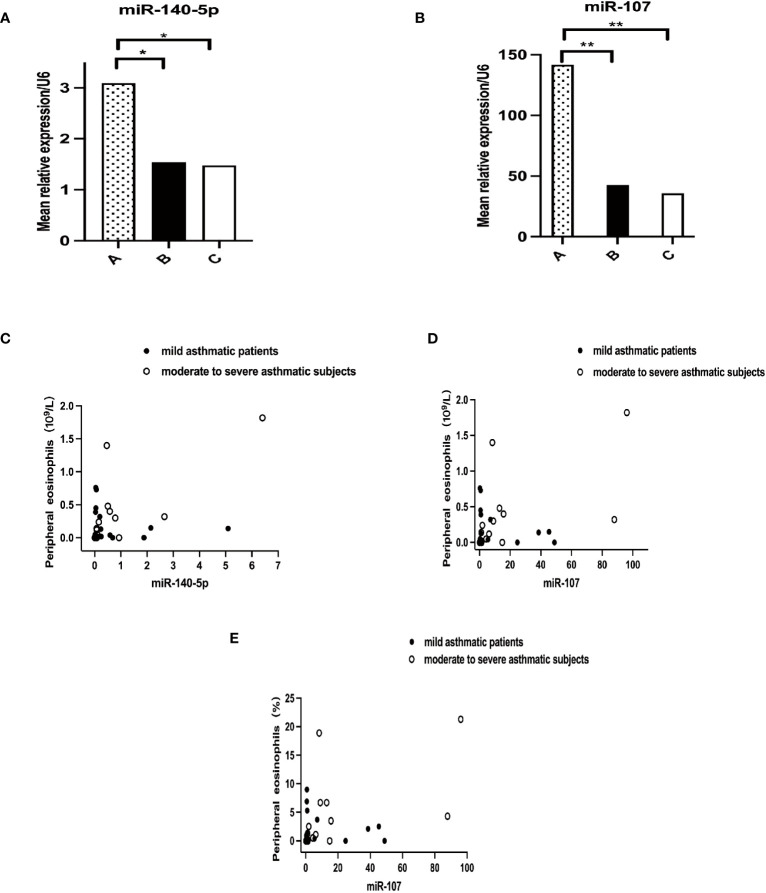
Correlation of miRNAs with peripheral blood eosinophils. A+B Mean relative expression of miRNA levels in three groups, **(A)** for miR-140-5p (p<0.0494) and **(B)** for miR-107 (p<0.0026). A–C in **Figure 1** representing healthy control group, mild asthmatic patient group and moderate to severe asthmatic subject group respectively. C+D Correlation analysis of miRNAs with absolute value of peripheral blood eosinophils. **(C)** for miR-140-5p (p=0.0016) and **(D)** for miR-107 (p=0.0185). **(E)** Correlation analysis of miR-107 with percentage of peripheral blood eosinophils. (p=0.0301). *p<0.05; **p<0.01.

Pairwise comparison by Kruskal-Wallis test and correlation analysis utilizing linear correlation regression were performed among the three groups. The results indicated that mean relative expressions of miR-140-5p and miR-107 level of asthma patients were significantly lower than those in healthy controls (*P*=0.0494 and 0.0026, respectively) (**Figures 3A, B**). Nevertheless, there were no statistical significance in these two miRNAs between asthma groups. The expression of miR-140-5p was positively correlated with the absolute value of peripheral eosinophils (r=0.7400, p=0.0016), and expression of miR-107 was closely relatively to the absolute value of peripheral eosinophils (r=0.5984, p=0.0185) and percentage of peripheral eosinophils (r=0.5594, p=0.0301). The correlation between expressions of miR-140-5p and miR-107 and other clinical indexes were shown in **Figures 3C-E**.

### The diagnostic efficacy of miR-140-5p and miR-107 for asthma

**Figure 4:**
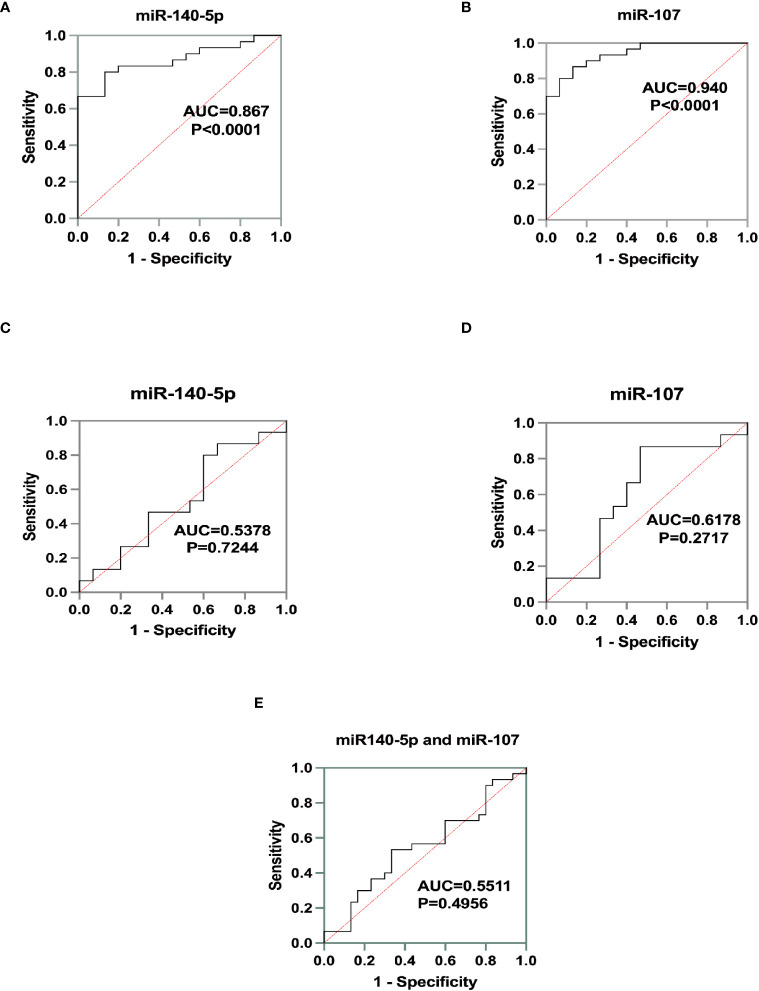
Identification of miRNA biomarker performance. A+B Receiver operating characteristics (ROC)curve for the test group as a measurement for asthma. **(A)** for miR-140--5p (AUC=0.8667, p<0.0001) and **(B)** for miR-107 (AUC=0.9400, p<0.0001). C+D ROC curve for the cohort as a measure for asthma severity. **(C)** for miR-140-5p (AUC=0.5378, p=0.7244) and **(D)** for miR-107(AUC=0.6178, p=0.2717). **(E)** Diagnostic efficacy of miR-140-5p and miR-107 for asthma severity by ROC curve. (AUC=0.5511, p=0.4965).

We used ROC curve to assess the diagnostic performance of miR-140-5p and miR-107 in asthma. It was demonstrated that miR-140-5p and miR-107 have good diagnostic efficacy for asthma (AUC= 0.8667 and 0.9400 shown in **Table 1**, **Figures 4A, B**) with good sensitivity (0.8000 and 0.8667 respectively) and specificity (0.8667 and 0.8667), while these two miRNAs have no performance in prediction of the severity of disease (AUC=0.5378 and 0.6178, shown in **Table 2**, **Figures 4C-E**).

**Table 1:** MiR-140-5p and miR-107 as diagnostic biomarkers during asthma attack.

biomarker	AUC value	P value	Sensitivity	Specificity	Optimal cutoff point
miR-140-5p	0.8667	<0.0001**	0.8000	0.8667	0.8591
miR-107	0.9400	<0.0001**	0.8667	0.8667	40.44
Characteristics	Mild asthma	Moderate to severe asthma	P value		
Eosinophil count (10^9^ cells/L)	0-0.76	0-1.82	0.7974		
Eosinophil percentage (%)	0-6.9	0-21.3	0.6737		

** p<0.001.

**Table 2:** MiR-140-5p and miR-107 as diagnostic biomarkers in mild asthma group and. moderate to severe asthma group.

biomarker	AUC value	P value	Sensitivity	Specificity	Optimal cutoff point
miR-140-5p	0.5378	0.7244	0.8	0.4	0.07073
miR-107	0.6178	0.2717	0.8667	0.5333	1.042
miR-140-5p、miR-107	0.5511	0.4965	0.5333	0.6667	0.9148

## Discussion

In our current study, we explored miRNA expression profiles of peripheral blood from healthy control and asthma patients. Significant differences were found in the expression of multiple miRNAs between the groups.

In the asthma group, the expressions of miR-6785-5p, miR-4428, miR-6893-5p, miR-513c-5p, miR-513b-5p, miR-5691, miR-512-3p, miR-4516, miR-8078 were remarkably elevated, while the expressions of miR-374a-5p, miR-26b-5p, miR-374b-5p, miR-223-3p, miR-20a-5p, miR-20b-5p, miR-140-5p, miR-107, miR-17-5p, miR-195-5p, miR-15b-5p were significantly reduced by miRNA microarray. The expressions of miR-107 and miR-140-5p were validated by Real Time PCR assay. Moreover, miR-107 and miR-140-5p are likely involved in the pathophysiology in asthma through regulating LIGHT gene.

Based on our previous research (6), LIGHT has been found to play a vital role in regulating the interactions between airway inflammation and remodeling in asthma. Many studies showed that miRNAs targeting asthma-susceptibility genes reflect the corresponding effect on the pathophysiology in asthma (7–9). Several studies have identified aberrant miRNA expression profiles associated with asthma severity (10), or therapy responses (11–13).

As a miRNA target gene, LIGHT may be regulated by miR-512-3p, miR-513b-5p, miR-5691, miR-107, miR-140-5p, and miR-17-5p screened by TargetScan. And then the normalized levels of these six miRNAs were revalidated through Real-Time PCR. It was demonstrated that the change trend in the miRNA expression patterns in asthma patients was similar to that in the miRNA microarray, and the same change patterns were obtained by in creasing the sample size for verification.

To investigate the candidate regulatory mechanism of the top two miRNAs (miR-107 and miR-140-5p) targeting LIGHT in asthma, we conducted GO functional and KEGG pathway enrichment analysis to investigate the target gene-miRNA network. Our findings indicated that the target genes have potential roles in the physiological processes of asthma, such as stem cell regulation and cytophagocytosis, through downstream signaling pathways (eg. PI3K-Akt, AMPK). There is emerging evidence that damaged cells can be rejuvenated by stem cells through mitochondrial transfer. Earlier studies found that epithelial mitochondrial dysfunction is critical in asthma pathogenesis (14). Recent studies revealed that miR-107 and miR-140-5p were characterized as tumor suppressors (15–18), and reduced in a various type of cancer. Additionally, the level of miR-140-5p was downregulated in the respiratory epithelium of patients with cystic fibrosis (19). MiR-140 suppresses airway inflammation and inhibits bronchial epithelial cell apoptosis in asthma by targeting GSK3β (20). MiR-107 inhibits airway smooth muscle cells migration by targeting Cdk6 (21), which was consistent with our results that miR-107 was significantly downregulated in asthma patients. Akt signaling pathway has been known to be involved in AMPK-dependent autophagic cell death, cell proliferation and differentiation.

According to a study on asthma, AMPK/PI3K/Akt pathway played a crucial role in promoting airway inflammation and lung injury in asthma model (22). Further studies are required to reveal the crosstalk between miRNAs and AMPK/PI3K/Akt signaling pathway in asthma.

Statistical tools were utilized to analyze the correlation between the expression level of miR-107 and miR-140-5p and various common clinical parameters. The results showed that the relative expressions of miR-107 and miR-140-5p were both positively correlated with the absolute peripheral eosinophil counts. Moreover, a significant correlation was also seen between miR-107 expression levels and the percentage of peripheral eosinophils. It was implied that the post-transcriptional regulators, miR-107 and miR-140-5p, may be involved in eosinophilic inflammation by negatively regulating LIGHT gene, and thus alleviate asthma symptoms. We also found that the relative expressions of miR-107 and miR-140-5p can be used as diagnostic biomarkers to distinguish patients with asthma from healthy control, whereas the diagnostic performance has not been established for asthma severity.

There are several limitations of this study that deserve mention. First, this is a single-center study with a small sample size, and a larger sample size will give us more accurate results. Second, there is lack of research on the serum levels of LIGHT, AMPK, PI3k and Akt, which could provide more information for the roles of the miRNAs in asthma progression. Third, further researches are needed to assess the clinical values of miR-107 and miR-140-5p in asthma, and to establish the validity and reliability of our findings.

## Conclusions

In summary, our study showed that miR-107 and miR-140-5p were differentially expressed between healthy individuals and asthmatics, and significantly correlated with clinically parameters including the number and percentage of peripheral eosinophils. These differently expressed miRNA profiles may take part in asthma pathogenesis by negatively regulating LIGHT, and act as biomarkers to identify asthma from healthy normal, while not useful in monitoring disease severity. Although the regulatory mechanism of miR-107 and miR-140-5p is yet unknown, they may serve as a potential biomarker for reflecting the pathology of asthma so as to guide the diagnosis.

## Data availability statement

The original contributions presented in the study are included in the article/**Supplementary Materials**, further inquiries can be directed to the corresponding authors.

## Ethics statement

The studies involving humans were approved by ethics committee of Shenzhen People’s Hospital. The studies were conducted in accordance with the local legislation and institutional requirements. The participants provided their written informed consent to participate in this study.

## Author contributions

GH: Visualization, Writing – original draft, Writing – review & editing. YD: Formal analysis, Methodology, Writing – review & editing. MX: Conceptualization, Data curation, Software, Validation, Writing – review & editing. RC: Resources, Supervision, Writing – review & editing. FS: Funding acquisition, Project administration, Supervision, Writing – review & editing.
