# Supplementary material for: Circulating miRNAs act as potential biomarkers for asthma
Source: Front Immunol. 2023 Dec 19;14:1296177. doi: 10.3389/fimmu.2023.1296177 (PMC10762778; doi:10.3389/fimmu.2023.1296177)
Supplement: Supplementary file 1 [file Table_1.docx]

**Table S1. Routine data of three groups of subjects(x±s)**

| Group | Cases | Gender (Male/Female) | Age（years of old） |
| --- | --- | --- | --- |
| Mild asthmatic patients | 5 | 2/3 | 48.69 ± 11.91 |
| Moderate to severe asthmatic subjects | 5 | 3/2 | 56.20 ± 13.27 |
| Healthy control subjects | 5 | 2/3 | 41.20 ± 11.52 |
| P value |  | 0.765 | 0.171 |
