# Supplementary material for: Circulating miRNAs act as potential biomarkers for asthma
Source: Front Immunol. 2023 Dec 19;14:1296177. doi: 10.3389/fimmu.2023.1296177 (PMC10762778; doi:10.3389/fimmu.2023.1296177)
Supplement: Supplementary file 2 [file Table_2.docx]

**Table S2. Routine data of three groups of subjects(x±s)**

| Group | Cases | Gender (Male/Female) | Age（years of old） |
| --- | --- | --- | --- |
| Mild asthmatic patients | 15 | 5/10 | 46.07 ± 11.88 |
| Moderate to severe asthmatic subjects | 15 | 5/10 | 49.33 ± 15.87 |
| Healthy control subjects | 15 | 6/9 | 49.33 ± 12.16 |
| P value |  | 0.913 | 0.745 |
