# Supplementary material for: Circulating miRNAs act as potential biomarkers for asthma
Source: Front Immunol. 2023 Dec 19;14:1296177. doi: 10.3389/fimmu.2023.1296177 (PMC10762778; doi:10.3389/fimmu.2023.1296177)
Supplement: Supplementary file 3 [file Table_3.docx]

**Table S3. Characteristics of the asthma groups**

| Characteristics | Mild asthma | Moderate to severe asthma |
| --- | --- | --- |
| Gender(male/female) | 5/10 | 5/10 |
| Age(years) | 46.07 ± 11.88 | 49.33 ± 15.87 |
| Neutrophil count (10^9^ cells/L) | 3.35-7.64 | 3.85-11.36 |
| Neutrophil percentage (%) | 47.3-87.5 | 44.9-86 |
| Lymphocyte count (10^9^ cells/L) | 0.43-3.78 | 0.75-2.72 |
| Lymphocyte percentage (%) | 5.4-35.3 | 7.8-27.7 |
| FEV1/FVC | 42.9-83.27 | 37-80 |
