# Supplementary material for: Circulating miRNAs act as potential biomarkers for asthma
Source: Front Immunol. 2023 Dec 19;14:1296177. doi: 10.3389/fimmu.2023.1296177 (PMC10762778; doi:10.3389/fimmu.2023.1296177)
Supplement: Supplementary file 4 [file Table_4.docx]

**Table S4. Upregulated miRNAs in asthmatic group**

| miRNAs | Fold change | P value |
| --- | --- | --- |
| miR-6785-5p | 7.95 | 0.000983 ^**^ |
| miR-4428 | 6.29 | 0.000451 ^**^ |
| miR-6893-5p | 5.97 | 0.001106 ^*^ |
| miR-513c-5p | 4.94 | 0.001883 ^*^ |
| miR-513b-5p | 4.27 | 0.002189 ^*^ |
| miR-5691 | 3.59 | 0.001670 ^*^ |
| miR-512-3p | 3.03 | 0.003383 ^*^ |
| miR-4516 | 3.03 | 0.000806 ^**^ |
| miR-8078 | 3.02 | 0.006030 ^*^ |

^*^ P<0.05; ^**^ P<0.001
