# Supplementary material for: Circulating miRNAs act as potential biomarkers for asthma
Source: Front Immunol. 2023 Dec 19;14:1296177. doi: 10.3389/fimmu.2023.1296177 (PMC10762778; doi:10.3389/fimmu.2023.1296177)
Supplement: Supplementary file 5 [file Table_5.docx]

**Table S5. Downregulated miRNAs in asthmatic patient group**

| miRNAs | Fold change | P value |
| --- | --- | --- |
| miR-374a-5p | 19.07 | 0.000132^**^ |
| miR-26b-5p | 16.22 | 0.000961^**^ |
| miR-374b-5p | 16.07 | 0.000258^**^ |
| miR-223-3p | 15.82 | 0.000529^**^ |
| miR-20a-5p | 15.49 | 0.001243^*^ |
| miR-20b-5p | 14.75 | 0.001037^*^ |
| miR-140-5p | 13.43 | 0.000612^**^ |
| miR-107 | 11.74 | 0.000636^**^ |
| miR-17-5p | 10.64 | 0.001250^*^ |
| miR-195-5p | 10.46 | 0.002387^*^ |
| miR-15b-5p | 10.23 | 0.001147^*^ |

^*^ P<0.05; ^**^ P<0.001
