# Supplementary material for: Circulating miRNAs act as potential biomarkers for asthma
Source: Front Immunol. 2023 Dec 19;14:1296177. doi: 10.3389/fimmu.2023.1296177 (PMC10762778; doi:10.3389/fimmu.2023.1296177)
Supplement: Supplementary file 6 [file Table_6.docx]

**Table S6. MiRNAs targeting LIGHT predicted by TargetScan software**

| miRNAs | Context ++ score | Context ++ score percentile |
| --- | --- | --- |
| miR-512-3p | -0.10 | 79 |
| miR-513b-5p | -0.03 | 45 |
| miR-5691 | -0.06 | 65 |
| miR-107 | -0.06 | 60 |
| miR-140-5p | -0.24 | 93 |
| miR-17-5p | -0.02 | 58 |

The value of context ++ score and context ++ score percentile represented a score of the probability of target genes predicted by TargetScan software. The lower score of the former and the upper score of the latter were likely the target genes.
