# Supplementary material for: Circulating miRNAs act as potential biomarkers for asthma
Source: Front Immunol. 2023 Dec 19;14:1296177. doi: 10.3389/fimmu.2023.1296177 (PMC10762778; doi:10.3389/fimmu.2023.1296177)
Supplement: Supplementary file 7 [file Table_7.docx]

**Table S7. Comparation fold change of miRNA expression level between miRNA microarray and Real-Time PCR**

| miRNAs | microarray of miRNA | Real-Time PCR |
| --- | --- | --- |
| miR-513b-5p | 4.27 | 1.18 |
| miR-512-3p | 3.03 | 0.62 |
| miR-140-5p | 13.43 | 2.49 |
| miR-17-5p | 11.74 | 1.64 |
| miR-107 | 10.64 | 2.15 |
